# ME/CFS and Post-Exertional Malaise among Patients with Long COVID

**DOI:** 10.3390/neurolint15010001

**Published:** 2022-12-20

**Authors:** Leonard A. Jason, Joseph A. Dorri

**Affiliations:** Center for Community Research, DePaul University, Chicago, IL 60614, USA

**Keywords:** ME/CFS, COVID-19, case definition, Long COVID

## Abstract

This study sought to ascertain the prevalence of Myalgic Encephalomyelitis/Chronic Fatigue Syndrome (ME/CFS) among a sample of 465 patients with Long COVID. The participants completed three questionnaires: (1) a new questionnaire measuring both the frequency and severity of 38 common symptoms of COVID and Long COVID, (2) a validated short form questionnaire assessing ME/CFS, and (3) a validated questionnaire measuring post-exertional malaise. The population was predominantly white, female, and living in North America. The mean duration since the onset of COVID-19 symptoms was 70.5 weeks. Among the 465 participants, 58% met a ME/CFS case definition. Of respondents who reported that they had ME/CFS only 71% met criteria for ME/CFS and of those who did not report they had ME/CFS, 40% nevertheless did meet criteria for the disease: both over-diagnosis and under-diagnosis were evident on self-report. This study supports prior findings that ME/CFS occurs with high prevalence among those who have persistent COVID-19 symptoms.

## 1. ME/CFS and Post-Exertional Malaise among Patients with Long-COVID

Estimates have widely ranged regarding how many individuals have persistent symptoms post SARS-CoV-2 infection [1,2,3]. A meta-analysis consisting of 15 studies with an aggregate sample of 47,910, revealed that 80% of people infected with COVID-19 had one or more persistent symptoms post-infection [4]. Based on a meta-analysis of worldwide data conducted by Chen and colleagues [1], the prevalence of post-COVID-19 conditions at months one, two, three and four were 37%, 25% 32% and 49%, respectively. Huang et al. (2022) determined that at two years after COVID infection, 55% still had one or more symptoms. It is clear from the amalgam of studies that COVID-19 infection does result in a significant proportion of those who have been infected with SARS-CoV-2 having lingering symptoms.

There are varying names and definitions for these persistent symptoms, and for this study they will be referred to as Long COVID. The Centers for Disease Control and Prevention [5] indicate that the key symptom domains of Long COVID are (1) general symptoms, such as tiredness or fatigue, post-exertional malaise, and fever, (2) respiratory and heart symptoms, (3) neurological symptoms, (4) digestive symptoms, (5) other symptoms, consisting of joint or muscle pain, rash, and changes in menstrual cycles. In a study by Fernandez-de-las-Penas and colleagues [6], Long COVID was seen to consist of seven symptom domains, namely neurocognitive, autonomic, gastrointestinal, respiratory, musculoskeletal, psychological, and others. Davis et al. [7] found the most common symptoms after six months are fatigue, post-exertional malaise, and cognitive dysfunction. Other studies also have found fatigue as the most prevalent symptom, followed by loss of taste or smell, dyspnea, and headache [8]. Furthermore, Huang et al. [2] found that after two years of COVID-19 infection the most common symptoms were fatigue and muscle weakness. Using an exploratory factor analysis, with 299 patients with Long COVID, Jason and Dorri [9] found three factors: cognitive dysfunction, autonomic dysfunction, and post-exertional malaise. Several other factor analyses with Long COVID samples have found similar as well as different factors [10,11,12,13].

Some patients with Long COVID might meet the case definition for Myalgia Encephalomyelitis/Chronic Fatigue Syndrome (ME/CFS), which is composed of symptoms including post-exertional malaise, unrefreshing sleep, and cognitive impairment [14,15]. For example, in the study by Jason and Islam [16] of 359 patients with Long COVID, 49% met ME/CFS criteria. In another study of 140 participants with Long-COVID, Bonilla et al. [17] found that 43% met the criterial for ME/CFS; Twomey, et al. [18] found that 58.7% of those infected with COVID-19 meet ME/CFS criteria. Similarly, Mancini et al. [19] studied patients with unexplained dyspnea among those who had Long COVID for a mean of 8 months and found that 46% met the criteria for ME/CFS. Furthermore, Kedor et al. [20] studied 42 Long COVID patients and found that 45% met the criteria for ME/CFS. In contrast, in González-Hermosillo et al.’s [21] Long COVID sample, only 13% met criteria for ME/CFS.

Some of the limitations of the above studies include small sample sizes and unclear methods for determining the ME/CFS case definitions. In addition, none of the studies provided a more comprehensive measure of the cardinal symptom of ME/CFS, that being post-exertional malaise. It is also unclear whether self-report data collected from patients on their ME/CFS status matches their symptoms related to a ME/CFS case definition. The current study seeks to determine the proportion of those who meet the ME/CFS case definition as well as post-exertional malaise, using validated questionnaires to determine ME/CFS status and post-exertional malaise.

## 2. Methods

### 2.1. Data Collection and Participants

Four hundred and eighty participants were recruited predominantly from social media sites dedicated to COVID-19 and Long COVID communities and groups. Furthermore, emails requesting support in recruitment were sent to various practitioners and researchers. Inclusionary criteria were having had COVID-19 and able to read and write in English. An exclusionary criterion was being under the age of 18. This study was approved by the DePaul University Institutional Review Board (protocol # IRB-2022-590, entitled “The DePaul Symptom Questionnaire—COVID (DSQ-COVID) Study”).

### 2.2. Measures

Participants completed a questionnaire consisting of three instruments on the secure survey platform Research Electronic Data Capture (REDCap) [22].

### 2.3. DePaul Symptom Questionnaire-COVID (DSQ-COVID)

A new questionnaire consisting of 38 symptoms was created for this study (Center for Community Research, Chicago, IL, USA). The initial questions consist of demographic information, the method of diagnosis, date of initial symptom onset, COVID-19 variant, hospitalization, incubation, vaccination status, organ damage due to contracting COVID-19, and prior medical conditions. Following these questions, there was a list of COVID-19-related symptoms. This symptom list was created by identifying the most common symptoms across the research literature and feedback from patient communities. The questionnaire requested participants to report both the frequency and severity of each of 38 symptoms for the past month. The frequency of each symptom over the past month was assessed on a five-point Likert scale with 0 = none of the time, 1 = a little of the time, 2 = about half the time, 3 = most of the time, and 4 = all of the time. The severity of each symptom over the past month was rated on a five-point Likert scale, consisting of 0 = symptom not present, 1 = mild, 2 = moderate, 3 = severe, and 4 = very severe. All frequency and severity scores were standardized to a 100-point scale. Finally, the frequency and severity scores for each symptom were averaged to create one score per symptom and then multiplied by 25 to create a composite score for each symptom ranging from 0 to 100. At the end of this questionnaire, respondents were asked “Do you have what has been referred to as Chronic Fatigue Syndrome, Myalgic Encephalomyelitis”, to which participants could respond by selecting “Yes, already had this condition before I had COVID-19”, “Yes, I have this condition after I had COVID-19.”, or “No”.

### 2.4. DePaul Symptom Questionnaire-Short Form (DSQ-SF)

DSQ-Short Form (DSQ-SF) is a subset of 14 items of 54 questions from the DePaul Symptom Questionnaire (DSQ), which was developed in an effort to standardize symptom assessment and directly compare and contrast individuals who meet various ME/CFS case definitions. The DSQ was originally constructed to assess the symptom requirements of the Fukuda et al. [23] CFS case definition and the ME/CFS Canadian Consensus Criteria (CCC) [14]. The DSQ’s items have demonstrated strong test–retest reliability [24], content validity [25], internal consistency reliability [25], and the ability to distinguish individuals with ME and CFS from healthy controls, as well as from individuals with other chronic illnesses [25,26,27]. Strand et al. [28] found a sensitivity of 98% when comparing the agreement between a physicians’ diagnosis of ME/CFS using the Canadian Consensus Criteria [14] and the DSQ’s assessment. Furthermore, when compared with other instruments that assess fatigue and health-related functioning, the DSQ’s symptom rating system was not limited by ceiling effects [26].

The DSQ-SF allows investigators to use a small number of items (n = 14) to determine whether patients meet ME/CFS case definitions. Similar to the DSQ, the DSQ-SF uses the mean of the frequency and severity scores for each symptom rated over the past 6 months and linearly transforming it into a 100-point scale [29]. The DSQ-SF is an effective, brief screening tool for measuring symptoms of ME/CFS and can determine whether a person meets ME/CFS case definitions. The scale demonstrates the ability to differentiate individuals with ME/CFS from healthy controls and patients with Multiple Sclerosis.

### 2.5. DePaul Post-Exertional Malaise (PEM) Questionnaire (DSQ-PEM)

This scale developed by Cotler et al. [30] consists of the following five DSQ items which measure PEM items: “A dead, heavy feeling after exercise”, “Muscle weakness even after resting”, “Next day soreness after everyday activities”, “Mentally tired after the slightest effort”, and “Physically drained after mild activity”. These five DSQ PEM items assess the frequency and severity of PEM over a six-month timeframe. Frequency was rated on a 5-point Likert scale: 0 = none of the time, 1 = a little of the time, 2 = about half the time, 3 = most of the time, and 4 = all of the time. Participants rated each symptom’s severity over the past six months on a 5-point Likert scale: 0 = symptom not present, 1 = mild, 2 = moderate, 3 = severe, 4 = very severe. The DSQ has been shown to have good test–retest reliability, and these five DSQ PEM items have good internal reliability (α = 0.84) [24]. Three additional PEM items within the DSQ examined duration of symptom exacerbation after activity. Participants were initially asked two questions: “Do you experience a worsening of your fatigue/energy related illness after engaging in minimal physical effort” and “Do you experience a worsening of your fatigue/energy related illness after engaging in mental effort”. They were then presented with a question measuring PEM duration: “If you feel worse after activities, how long does this last”. Participant responses of PEM duration were coded as: 0 = Not having a problem with energy/fatigue, 1 = 1 h or less, 2 = 2–3 h, 3 = 4–10 h, 4 = 11–13 h, 5 = 14–23 h, and 6 = More than 24 h. Good test–retest reliability has been found for both branching logic questions of symptom exacerbation due to physical activity (k = 0.84) and symptom exacerbation due to mental activity (k = 0.74) [24]. The fourth supplementary PEM item assessed how quickly patients would recover from activities that are typically undertaken by healthy individuals, asking “If you were to become exhausted after actively participating in extracurricular activities, sports, or outings with friends, would you recover within an hour or two after the activity ended?” This item was previously demonstrated as having good test–retest reliability (k = 0.88) [24]. The fifth supplementary PEM item assessed whether participants were not exercising because it made their symptoms worse. Participants were asked “If you do not exercise, is it because exercise makes your symptoms worse?” This item was previously demonstrated as having good test-retest reliability (k = 0.79) [24].

### 2.6. Outcome Measure of Functional Impairment

Patients also self-reported on their level of impairment using a 7-point Likert scale ranging from “I am not able to work or do anything, and I am bedridden” to “I can do all work or family responsibilities without any problems with my energy”.

### 2.7. ME/CFS Criteria

This case definition involves the CCC [14]. Participants needed to have experienced substantial reduction in educational, social, and personal activities, and this was assessed with the following question: “Since the onset of your problems with fatigue/energy, have your symptoms caused a 50% or greater reduction in your activity level?”. This question has shown to closely match other methods for measuring substantial reduction in functioning [31]. Participants needed to have the following symptoms: post-exertional malaise, unrefreshing sleep or disturbance of sleep quantity, pain, and two or more neurocognitive manifestations. Additionally, they needed to have at least one symptom from one of the following three categories: autonomic, neuroendocrine, or immunologic.

Outcome measure. Patients were also asked to respond to the following question: “Which statement best describes your fatigue/energy level over the last month?” with the following seven response options: “I am not able to work or do anything, and I am bedridden”, “I can walk around the house, but I cannot do light housework”, “I can do light housework, but I cannot work part-time”, “I can only work part-time at work or on some family responsibilities”, “I can work full time, but I have no energy left for anything else”, “I can work full time and finish some family responsibilities, but I have no energy left for anything else”, and “I can do all work or family responsibilities without any problems with my energy”. On this 7-point scale, lower scores represent more severe impairment.

### 2.8. Replacing Missing Values

Participants missing 10% or more of items from the DSQ-COVID were removed from analyses. For those remaining participants, missing values were replaced using a method dependent on the nature of the missing value. Participants could have missing data for either the frequency, the severity, or for both dimensions of a symptom. When a participant reported a “0” for either the frequency or the severity of a symptom (but not both), the corresponding score was replaced with a “0”. The reasoning is that a symptom should occur “none of the time” (frequency = 0) if the symptom is “not present” (severity = 0). Next, if a participant reported a frequency or severity score greater than “0” for a symptom but did not report a corresponding frequency or severity score, every case from the total sample that matched the participant’s reported score was reviewed and used to calculate the mode of the corresponding scores. The mode of the corresponding scores was used to replace the participant’s missing value. Finally, if a participant had missing scores for both frequency and severity of a symptom, both missing scores were replaced with the overall median scores for that symptom among the rest of the population.

### 2.9. Statistical Analyses

IBM SPSS version 28 was used for analyses. The sample was divided into those who met ME/CFS criteria and those who did not. Next, chi-square and *t*-test statistics were conducted to assess for differences between these groups across key demographic variables, namely age, sex, duration since initial symptom onset, ethnicity, and region. Variables that were statistically significant were controlled in subsequent analyses comparing the two groups. 

## 3. Results

After removing those participants who completed less than 90% of the survey, 465 participants remained. Among them, 272 (58%) met the CCC case definition for ME/CFS and 193 (42%) did not. Duration, sex, and region were statistically significant between the two groups (See Table 1) and they were used as control variables in the analyses below.

For Table 2, Table 3 and Table 4, “Symptom Means” refer to a 100-point composite score of frequency and symptoms, with higher numbers signifying more burden. Those meeting CCC criteria had directionally higher mean scores for every symptom on the DSQ-COVID and their difference were statistically significant for 36 of the 38 symptoms (see Table 2). Similarly, those meeting CCC criteria had higher mean scores for every symptom on the DSQ-SF and the differences between each group were statistically significant across all symptoms (see Table 3). Furthermore, those meeting CCC criteria had higher mean scores for every symptom on the PEM questionnaire and their differences were statistically significant across all (See Table 4). The first five DSQ PEM items assess the frequency and severity of PEM, whereas the second five items are asked in a different way, as indicated in the Methods section.

On the outcome measure of functional impairment, with lower scores representing more severe impairment, those with ME/CFS scored a mean of 3.24 (SD = 1.17), which was significantly lower than those not meeting ME/CFS criteria (M = 4.10; SD = 1.51), *t*(461) = 6.89, *p* < 0.001). All participants who meet CCC criteria had a 50% or greater reduction in their energy level, which was expected as it is part of the criteria.

Finally, discrepancies between patient self-reports of having or not having ME/CFS and meeting versus not meeting the ME/CFS case definition is reflected in Table 5, and considerable misreporting is evident. Of respondents who reported that they had ME/CFS (187 + 75 = 262), only 71.37% (N = 187) met criteria for ME/CFS and of those who did not report they had ME/CFS (78 + 117 = 1 95), 40.00% (N = 78) nevertheless did meet criteria for the disease.

## 4. Discussion

This study confirms prior research showing that a substantial percentage of people who contract COVID-19 develop a condition that meets the criteria for a CCC diagnosis of ME/CFS. This percentage is comparable to earlier studies [16,17,18,20], except that of González-Hermosillo et al. [21]. It is apparent that many who have been infected with SARS-CoV-2 and have persisting symptoms will meet a ME/CFS case definition.

This study also found that 58% of those with persisting COVID-19 symptoms met ME/CFS criteria. Furthermore, of those who reported that they had ME/CFS, only 71% met criteria for ME/CFS and of those who did not report they had ME/CFS, 40% nevertheless did meet criteria for the disease. This is a serious issue for investigators to consider as they assess patients for this comorbid condition. In adult and pediatric community-based epidemiologic studies, researchers have found that from 90 to 95% of patients with ME/CFS do not know they have ME/CFS [32,33]. Based on these findings and the current study, due to participants not knowing the symptoms of ME/CFS, it is not sufficient to just ask patients whether or not they have ME/CFS, as most have no idea of what symptoms are in the established ME/CFS case definitions. In addition, asking the participants whether they had been diagnosed with ME/CFS has problems in that many health care practitioners have difficulties making accurate diagnoses as they are not familiar with established ME/CFS case definitions. As has been learned by ME/CFS researchers, using psychometrically sound instruments with good reliability and validity is key to determining whether patients meet ME/CFS case definitions [34].

Symptoms with the highest mean scores across DSQ-SF for those meeting ME/CFS criteria were: Minimum exercise makes you physically tired, Fatigue/extreme tiredness; Feeling unrefreshed after you wake up in the morning; Next day soreness or fatigue after non-strenuous, everyday activities,; Difficulty paying attention for a long period of time; and Problems remembering things. These are all cardinal symptoms of ME/CFS and three of these symptoms were PEM, two involved cognitive impairment, and one involved unrefreshing sleep. Those COVID-19 patients who meet ME/CFS criteria were more symptomatic on all COVID-19 symptoms. It is a tautology to show that ME/CFS symptoms were more common in people diagnosed with ME/CFS, but more importantly, it is interesting that some Long COVID symptoms (e.g., loss of or change in smell and/or taste) also were more frequent and severe in people who met diagnostic criteria for ME/CFS. 

This is the first Long COVID study to use the DSQ-PEM, and PEM has been a key component of ME/CFS for over three decades [35,36]. These items have shown great reliability to identify PEM in patients with ME/CFS [30]. Identifying PEM in Long COVID patients early on can inform better diagnosis, which can include preparing one’s living and work conditions for worsening of symptoms. This can include making their living space more accessible, requiring less exertion to accomplish basic daily tasks, and arranging adjustments to work conditions, transportation, schedule of hours, and potential changes in finances.

There are some limitations in this study. Firstly, the population primarily consists of females (the female/male ratio among the respondents was much higher than in most studies) and the sample was primarily white. In addition, the data are cross sectional and more long-term data are needed to determine whether the sample population continues to have ME/CFS symptoms over time. Another weakness of the study is that the study subjects were recruited from online patient communities of people whose “COVID-19” was diagnosed in various ways. In addition, another limitation is that there was no assessment of multiplicity correction for the analyses. The authors recommend that only findings at the 0.01 level be considered reliable, as at this level, only one significant comparison in one hundred might have occurred by chance. Finally, all available data were not explored in this study, including COVID-19 variant, hospitalization, incubation, and vaccination status, as well as whether the results were influenced by the method of diagnosis (e.g., whether PCR-positive or not) and COVID-19 variant. Likewise, the questionnaires distinguished whether study respondents had developed ME/CFS before vs. after developing COVID-19. These are all important issues, but they will be addressed in future publications as one article cannot describe all the findings from this very large data set.

We believe that there is a need to study the relation of COVID-19 and ME/CFS, and, in particular, a key symptom of ME/CFS, that of post-exertional malaise. Studying these illnesses simultaneously can provide greater insight into their development, trajectory, and treatment. One example of this is to screen for ME/CFS among Long COVID patients in government-funded initiatives to study COVID-19. Furthermore, in clinical settings, practitioners can incorporate the DSQ-SF and PEM questionnaires as pre-screeners for each visit or periodically. The benefit of these questionnaires is that they are relatively short and reliable, making them ideal for those suffering from cognitive and exertional symptoms that persist in these populations. Furthermore, the ability to administer these validated questionnaires online benefits patients by reducing the resources needed to commute, take time off work, and of course the worsening of symptoms as a result of exertion in travelling to practitioners and medical care centers. The continued infections globally of SARS-CoV2, the development of Long COVID, and the prevalence of ME/CFS among these populations, require researchers to collaborate on quickly measuring the right symptoms and correctly identifying those with various long-term chronic fatigue illnesses, so as to mitigate the burden of this disease for decades to come.

## Figures and Tables

**Table 1:** Demographic information across the two groups of those who meet CCC criteria and those who do not meet CCC criteria.

	Meets CCC	Does Not Meet CCC	Sig
	*M* (SD)	*M* (SD)	
Age	48.47 (11.87)	46.54 (12.61)	
Duration (weeks)	77.67 (34.66)	60.61 (42.76)	**
	% (n)	% (n)	
Sex			
Female	86.40 (235)	76.68 (148)	**
Male	8.82 (24)	17.10 (33)	
Other	4.41 (12)	5.18 (10)	
Ethnicity			
White	92.65 (252)	92.23 (178)	
Asian	1.10 (3)	3.63 (7)	
Hispanic or LatinX	1.10 (3)	2.59 (5)	
Black	0.37 (1)	0.52 (1)	
Other	4.79 (13)	1.04 (2)	
Region			**
North American	40.81 (111)	85.49 (165)	
Europe	45.96 (125)	2.59 (5)	
Other	11.76 (32)	8.29 (16)	

** *p* < 0.01.

**Table 2:** DSQ-COVID symptom means and standard deviations.

	Meets CCC	Does Not Meet CCC	Sig
	*M* (SD)	*M* (SD)	
Symptoms that get worse after physical or mental activities (also known as post-exertional malaise)	79.08 (16.76)	56.67 (27.44)	**
Fatigue/extreme tiredness	78.09 (14.24)	58.06 (22.26)	**
Difficulty thinking and/or concentrating	66.45 (18.75)	39.54 (22.92)	**
Sleep problems	60.20 (26.20)	45.28 (26.40)	**
Muscle aches	58.49 (25.25)	37.59 (27.80)	**
Memory loss	58.36 (22.05)	29.81 (22.80)	**
Bone and/or joint pain	53.55 (27.44)	35.28 (29.45)	**
Headache	52.37 (24.25)	38.15 (20.05)	**
Feeling faint, dizzy, and/or difficulty thinking soon after standing up from a sitting or lying position	49.93 (27.44)	30.00 (22.35)	**
Changes in desire for, comfort with or capacity for sex	51.91 (34.68)	30.06 (33.96)	**
Stress	50.92 (25.80)	38.42 (24.70)	**
Vision problems (blurry, light sensitivity, difficult reading or focusing, floaters, flashing light)	47.17 (26.84)	31.20 (26.93)	**
Gastrointestinal (belly) symptoms (pain, feeling full or vomiting after eating, nausea, diarrhea, constipation)	47.50 (28.53)	36.39 (26.29)	**
Palpitations, racing heart, arrhythmia, and/or skipped beats	41.71 (26.79)	30.19 (23.48)	**
Nerve problems (tremor, shaking, abnormal movements, numbness, tingling, burning, can’t move part of body, new seizures)	45.26 (29.22)	26.20 (26.71)	**
Anxiety	40.59 (30.20)	32.22 (25.48)	**
Shortness of breath and/or trouble breathing	41.64 (23.25)	29.44 (26.21)	**
Heavy legs and/or swelling of leg	37.76 (29.46)	22.50 (28.81)	**
Problems with hearing (hearing loss, ringing in ears)	40.53 (32.92)	29.44 (30.41)	*
Depression	37.17 (30.28)	26.57 (24.50)	**
Nose congestion	34.93 (25.67)	24.44 (23.25)	**
Fever, chills, and/or sweating	34.54 (26.23)	23.98 (24.29)	**
Dry eyes	35.39 (31.08)	21.67 (25.93)	**
Change in blood pressure	32.76 (30.79)	21.11 (24.79)	**
Pins and needles feeling	32.17 (26.83)	17.87 (22.37)	**
Chest pain	29.93 (23.24)	20.65 (23.67)	**
Dry skin/peeling	28.62 (29.03)	17.41 (24.05)	**
Loss of or change in smell and/or taste	24.41 (31.90)	14.72 (25.82)	*
Bladder problems (incontinence, trouble passing urine or emptying bladder)	24.93 (27.32)	11.76 (20.87)	**
Sore throat	21.84 (20.29)	16.48 (19.05)	*
Color changes in your skin such as red, white or purple	23.36 (26.88)	11.57 (20.18)	**
Skin rash	23.03 (28.28)	11.94 (20.37)	**
Sore tongue, mouth, and/or difficulty swallowing	22.17 (25.02)	13.80 (20.73)	*
Gynecological symptoms (e.g., change in menstruation or menopause)	22.96 (32.35)	13.33 (24.40)	*
Loss of hair	24.74 (29.09)	17.50 (24.82)	*
Cough	20.92 (22.64)	18.43 (22.69)	
Ear pain	19.34 (24.69)	11.48 (16.24)	**
Weight loss	9.54 (20.13)	10.00 (21.48)	

* *p* < 0.05, ** *p* < 0.01. Note symptoms are arranged in descending order of means for the group meeting CCC criteria. Variables duration, sex, and region are covariates.

**Table 3:** DSQ-SF symptom means and standard deviations.

	Meets CCC	Does Not Meet CCC	Sig
	*M* (SD)	*M* (SD)	
Minimum exercise makes you physically tired	82.50 (16.05)	54.35 (28.78)	**
Fatigue/extreme tiredness	81.84 (13.33)	56.48 (24.71)	**
Feeling unrefreshed after you wake up in the morning	80.26 (15.39)	48.70 (26.02)	**
Next day soreness or fatigue after non-strenuous, everyday activities	75.13 (18.68)	48.43 (27.42)	**
Difficulty paying attention for a long period of time	71.91 (17.00)	37.59 (24.79)	**
Problems remembering things	67.11 (21.21)	33.06 (24.36)	**
Pain or aching in your muscles	61.58 (25.21)	37.50 (29.14)	**
Feeling hot or cold for no reason	49.93 (27.35)	39.44 (22.12)	*
Irritable bowel problems	44.80 (31.02)	29.07 (27.57)	**
Feeling unsteady on your feet, like you might fall	44.34 (27.08)	23.89 (22.42)	**
Bloating	46.45 (29.39)	26.57 (27.34)	**
Cold limbs (e.g., arms, legs, hands)	45.33 (29.07)	23.98 (23.61)	**
Flu-like symptoms	36.71 (26.90)	22.50 (23.50)	**
Some smells, foods, medications, or chemicals make you feel sick	28.36 (30.04)	15.37 (21.38)	**

* *p* < 0.05, ** *p* < 0.01. Note symptoms are arranged in descending order of means for the group meeting CCC criteria. Variables duration, sex, and region are covariates.

**Table 4:** DSQ-PEM symptom means and standard deviations.

	Meets CCC	Does Not Meet CCC	Sig
	*M* (SD)	*M* (SD)	
Minimum exercise makes you physically tired	78.09 (18.88)	49.17 (29.70)	**
Physically drained or sick after mild activity	74.41 (18.91)	43.70 (29.61)	**
Next day soreness or fatigue after non-strenuous, everyday activities	72.96 (19.00)	44.72 (25.89)	**
Mentally tired after slightest effort	69.93 (20.41)	38.06 (24.71)	**
Dead, heavy feeling after starting to exercise	63.75 (29.14)	38.06 (32.48)	**
	% (n)	% (n)	
If you were to become exhausted after actively participating in extracurricular activities, sports, or outings with friends, would you recover within an hour or two after the activity ended?	3.32 (9)	20.83 (40)	**
Do you experience a worsening of your fatigue/energy related illness after engaging in minimal physical effort?	97.42 (264)	75.00 (144)	**
Do you experience a worsening of your fatigue/energy related illness after engaging in mental effort?	95.96 (261)	67.71 (130)	**
If you feel worse after activities, how long does this last?			**
1 h or less	0.74 (2)	6.04 (11)	
2–3 h	3.68 (10)	15.93 (29)	
4–10 h	11.76 (32)	15.93 (29)	
11–13 h	3.31 (9)	3.85 (7)	
14–23 h	7.72 (21)	11.54 (21)	
24 h or more	72.79 (198)	46.70 (85)	
If you do not exercise, is it because exercise makes your symptoms worse?	97.31 (253)	79.33 (142)	**

** *p* < 0.01. Note symptoms are arranged in descending order of means for the group meeting CCC criteria. Variables duration, sex, and region are covariates.

**Table 5:** Discrepancies between self-reports and ME/CFS classification.

	Self-Report ME/CFS	
	Yes	No
	% (n)	% (n)
Meet CCC Criteria		
Yes	70.57 (187)	29.43 (78)
No	39.06 (75)	60.94 (117)

Note: Several individuals did not indicate their ME/CFS status, so the numbers do not add up to 465.

## Data Availability

Contact the first author.
